# Impact of Market Competition on Continuity of Care and Hospital Admissions for Asthmatic Children: A Longitudinal Analysis of Nationwide Health Insurance Data 2009-2013

**DOI:** 10.1371/journal.pone.0150926

**Published:** 2016-03-09

**Authors:** Kyoung Hee Cho, Eun-Cheol Park, Young Soon Nam, Seon-Heui Lee, Chung Mo Nam, Sang Gyu Lee

**Affiliations:** 1 Department of Public Health, Graduate School, Yonsei University, Seoul, Korea; 2 Institute of Health Services Research, College of Medicine, Yonsei University, Seoul, Korea; 3 Department of Preventive Medicine, College of Medicine, Yonsei University, Seoul, Korea; 4 Department of Quality Assessment, Health Insurance Review & Assessment Service, Wonju, Korea; 5 Department of Nursing Science, College of Nursing, Gachon University, Seongnam, Korea; 6 Department of Biostatistics, College of Medicine, Yonsei University, Seoul, Korea; 7 Graduate School of Public Health, Yonsei University, Seoul, Korea; University of South Australia, AUSTRALIA

## Abstract

**Background:**

Ambulatory care-sensitive conditions, including asthma, can be managed with timely and effective outpatient care, thereby reducing the need for hospitalization.

**Objective:**

This study assessed the relationship between market competition, continuity of care (COC), and hospital admissions in asthmatic children according to their health care provider.

**Methods:**

A longitudinal design was employed with a 5-year follow-up period, between 2009 and 2013, under a Korean universal health insurance program. A total of 253 geographical regions were included in the analysis, according to data from the Korean Statistical Office. Data from 9,997 patients, aged ≤ 12 years, were included. We measured the COC over a 5-year period using the Usual Provider Continuity (UPC) index. Random intercept models were calculated to assess the temporal and multilevel relationship between market competition, COC, and hospital admission rate.

**Results:**

Of the 9,997 patients, 243 (2.4%) were admitted to the hospital in 2009. In the multilevel regression analysis, as the Herfindahl–Hirschman Index increased by 1,000 points (denoting decreased competitiveness), UPC scores also increased (ß = 0.001; p < 0.0001). In multilevel logistic regression analysis, the adjusted odds ratio (OR) for hospital admissions for individuals with lower COC scores (≥ 2 ambulatory visits and a UPC index score of < 1) was 3.61 (95% CI: 2.98–4.38) relative to the reference group (≥ 2 ambulatory visits and a UPC index score of 1).

**Conclusions:**

Market competition appears to reduce COC; decreased COC was associated with a higher OR for hospital admissions.

## Introduction

Ambulatory care-sensitive conditions (ACSCs), such as asthma, diabetes, hypertension, chronic heart failure, and chronic obstructive pulmonary disease, can be managed with timely and effective outpatient care, thereby reducing the need for hospitalization [1]. In particular, the prevalence of asthma among pediatric patients remains high in adulthood, unlike certain other chronic diseases. The socioeconomic cost of asthma, due to direct medical costs and indirect costs (work and school absences) was 4 trillion Won in 2004 [2]. In 2011, the hospitalization rate in Korea for patients with uncontrolled asthma was 102 per 100,000 population, more than two-fold the Organization for Economic Cooperation and Development (OECD) average of 52 per 100,000 population ([3]. Respiratory disease, including asthma, was the sixth leading cause of death in 2012, according to the National Statistics Office [4].

Policymakers worldwide are beginning to recognize the magnitude of the problem of efficiently managing chronic diseases with limited resources. High continuity of care (COC) should be provided to reduce the risk of complications [5], improve preventive care [6,7], increase patient satisfaction [8] and compliance [9,10], and decrease the requirement for emergency and inpatient medical services [11–16]. Particularly in primary care, COC is being introduced to improve care quality and cope with the increased workload associated with chronic diseases [17]. There are clear benefits conferred by COC; from the perspective of economic theory, a greater number of choices may increase the likelihood of matching patients to the most appropriate providers. Competition between providers could also improve the quality of services from the patient’s perspective [18]. The Korean system is markedly different from the managed care delivery of the United States, in which the patient’s choice of health care provider is regulated and restricted [19]. All Korean hospitals can provide outpatient services, and there are no barriers to using superior health services and patients can select health care providers without limitations. Moreover, all health care providers in South Korea are in competition. In the case of South Korea, NHI is the single insurer that manages the NHI program and the costs of medical service; hence health care providers do not compete in the perspective of cost but in the perspectives of providing amenities, friendly service, and detailed explaining in order to attract more patients. Thus Individual’s freedom of choice may improve quality of care[20]. However, in contrast this may cut off continuous care in the perspective of information flow and the relationship between health care providers and patients. This reduction in the continuity of care may lead to undesirable outcomes.

This study assessed the effect of competition among health care providers with regard to COC, and determined the association between COC and hospital admissions in asthmatic children.

## Methods

### Data sources

This study used three data sources. The first source was the National Health Insurance claims data of asthmatic children, obtained from the Health Insurance Review and Assessment (HIRA) between 2009 and 2013. This sample data are representative of the country as a whole, and is stratified according to sex, age (grouped according to 5-year intervals), and outpatient/inpatient diseases status. Information on 23,909 patients (approximately 1% of all Korean asthma patients) was available, including full medical histories between 2009 and 2013, age, sex, costs of care, prescription history, diagnostic test results, and several other parameters. The National Health Insurance Corporation represented the second source of data, and was used to calculate market concentration; this population data, for respiratory diseases between 2002 and 2012, did not include information pertaining to prescription history or diagnostic test results. The third data source was the Korean Statistical Information Service, which was used to gather information on regional health care providers. Ethical approval for this study was granted by the institutional review board of the Graduate School of Public Health, Yonsei University, Seoul, Korea. This study was retrospective research, and our data was not including personal information. So we could not obtain consent. As consent was not obtained, patient records and information was anonymized and de-identified prior to analysis.

### Study population

Data for 23,909 asthmatic patients were available for 2009. Of these, we excluded 13,912 patients: 13,372 patients were >13 years of age, 2 patients did not possess health insurance or medical aid (other health securities were in place, e.g., veteran-specific security), and 538 patients with a main diagnosis of J45 were not prescribed asthma medication, had taken no test for asthma, and made only 1 outpatient visit during the year.

**Fig 1:**
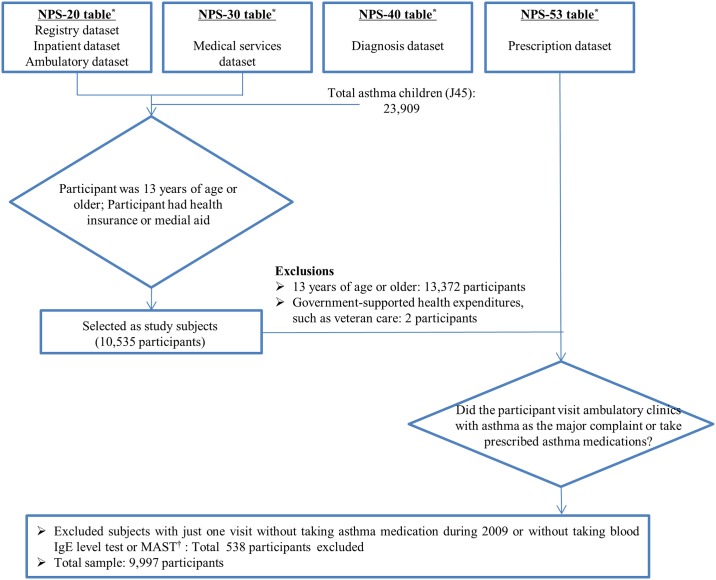
Flowchart of study participant selection. *, The National Health Insurance claims data consisted of 4 tables (20, 30, 40, and 53 table). Each table includes different information. ^†^, MAST; multiple radioallergosorbent test.

We defined medical usage as cases in which patients visited an outpatient clinic, received a main diagnosis of J45, and received a prescription for ≥ 1 asthma medication [21]. We excluded 538 asthma patients who made only 1 outpatient visit during the year and were not prescribed asthma medication. However, regardless of the number of outpatient visits, if the patient had undergone a test of IgE blood levels or multiple radioallergosorbent tests (MAST), they were eligible for inclusion. The rationale for this criterion was to improve the accuracy of asthma diagnoses, which were based on information obtained from the insurance claims database. Diagnosis accuracy using Korean claims data are approximately 70% [22]. Therefore, an additional criterion was required to ensure that all of the included participants were actually asthmatic; frequent asthma-related visits were used for this purpose, rendering a final study sample of 9,997 (Fig 1).

### Variables

The dependent variable was hospital admissions due to asthma, defined as using inpatient medical services for >1 day and receiving a main diagnosis of J45.

Between 2009 and 2013, we measured COC for each patient using the Usual Provider Continuity (UPC) index [23]. Three indices are commonly used to measure COC: the COC index, the Sequential Continuity of Care (SECON) index, and the UPC index. We selected the UPC index because it was the instrument most capable of identifying the association between market competition and COC. This index describes the proportion of visits made to the patient’s regular physician out of all visits. According to the literature, if no regular physician is assigned to a patient, the index is computed so that the physician that the patient visited most frequently during the defined time period is assigned as the patient’s regular physician. The index ranges between 0 and 1, with higher values indicating superior COC. The formula for the UPC index is as follows:

$$UPC\;=\;\frac{1\leq j\leq M(n_{j})}{N}$$

*N* is the total number of ambulatory care visits, *n*_*j*_ is the number of visits to the *j*th provider with whom the patient made the greatest number of visits, and *M* is the number of potentially available providers. In this study, potentially available providers refer to healthcare institutions and not to medical physicians. We arbitrarily categorized this into three groups: four or more ambulatory visits and a UPC index rating above mean, four or more ambulatory visits and a UPC index below the mean, and three or below ambulatory visits. We included in the study population subjects whose primary diagnosis was asthma and had a record of being prescribed with asthma medications. Regardless of ambulatory visits, we included only identified patients whose primary diagnosis was asthma and tested by related asthma diagnostic tests. In fact, we made an effort to identify real asthmatic children as in the case of children, even if a child makes only one ambulatory visit per year, he or she is likely to be a patient with asthma. In addition, although asthma is one of the ambulatory care-sensitive conditions (ACSCs), asthma shares many similar characteristics with other chronic diseases such as diabetes, hypertension and chronic obstructive pulmonary disease which is controlled through daily medication. Hence the total number of visits due to asthmatic symptoms, which can generally be controlled through medication, may indicate the severity of the disease itself; increased ambulatory visits can thus mean that the disease is severe. Therefore, we divided based on whether the number of ambulatory visits equaled or exceeded four, and then further divided depending on whether the UPC index score scored above the mean score of the group with four or more ambulatory visits.

We measured market competition among health care providers using the Herfindahl–Hirschman Index (HHI). To calculate the HHI, we distinguished hospital markets according to 253 administrative districts within Korea. The equation for HHI is as follows:

$$Herfindahl-Hirschman\;Index\;(HHI)\;=\;\sum_{i\;=\;0}^{n}S_{i}^{2}$$

The HHI is the sum of squared market shares for hospitals within a market. Market share (*S*_*i*_) is computed using total charges for patients with respiratory diseases excluding lung cancer; *i* denotes an individual hospital, and *n* is the total number of hospitals in the market. A low HHI indicates the presence of less-dominant hospitals or greater competition in that particular market. Conversely, a high HHI indicates the presence of dominant hospitals or reduced competition [24,25]. To examine the relationship between market competition and hospital admission rate, we divided HHI into four categories. The total of number of patient visits was 8,202,548,109 for outpatient clinics (it is probable for one individual to utilize the facilities multiple times) nationwide during 2009 for respiratory-related diseases, excluding lung cancer, with a total of 17,029 outpatient clinics included in the HHI analysis. Community clinics were included in 17,029 outpatient clinics because community clinics shared a substantial number of patients in small areas.

### Covariates

Covariates comprised both individual and regional variables. Individual variables included age, sex, insurance type (e.g., health insurance, medical aid), Charlson Comorbidity Index (CCI) score (0–3^+^), type of main visiting medical institution (general or tertiary hospital, hospital, clinic, public health center), total ambulatory care visits (1–4^+^), use of inhaled corticosteroid, and presence of respiratory distress. We used three proxy variables: the CCI score of patient severity, presence of respiratory distress, and total ambulatory care visits. For the CCI, only the comorbidity component was calculated [26]. All diagnostic information was collected using inpatient and outpatient billing data for each year. Respiratory distress was defined as a main diagnosis of J45, with R06 (on the ICD-10) as an additional diagnosis. The type of main visiting institution was the healthcare institution visited most frequently for outpatient care. Regional variables included inpatient beds per 1,000 population, total number of hospitals, physicians per 1,000 population, and HHI. Regional variables were categorized according to administrative district; Si and Do (metropolitan and state; *n* = 16); and Si, Gun, and Gu (city, county, and ward; *n =* 253). Each Si and Do has elements of Si, Gun, and Gu. We measured regional variables at the Si, Gun, and Gu levels. We repeatedly measured all individual and regional variables between 2009 and 2013.

### Statistical analysis

We initially conducted a descriptive study analyzing baseline (2009) frequencies and means. To identify the association between market competition and COC, and to allow for non-independence between study subjects clustered within districts, we fitted a random intercept model using a longitudinal technique and taking time-invariant characteristics into account. To assess the association between COC and hospital admissions, we fitted a random intercept logistic regression model, adjusted for individual and regional level variables. Since our study sample has a nested data structure where individual levels are clustered within higher-level regions, hierarchical linear modeling is used to examine the relationships between hospital admission and its covariates. Two-level random effects models are fitted using the maximum likelihood estimation (MLE), in order to capture associations between individual and regional level measures and the dependent variable. The basic model for the multilevel analysis is represented by the following equation:

$$Y_{ij}\;=\;\beta_{0}+\beta_{1}R_{ij}+\;\beta_{2}R_{j}+\beta_{3}X_{ij}+\;\mu_{j}+\varepsilon_{ij}$$

*Y*_*ij*_ refers to hospital admission for individual I (Level 1) in region j (Level 2), *R*_*ij*_ the set of main independent variables measured at Level 1, *R*_*j*_ the Level-2 variables, and X the vectorfor control variables. The *β*’s are fixed parameters to be estimated, *μ*_*j*_ the region-specific random effect, and *ε*_*ij*_ a component of the error term (Raudenbush and Bryk, 2002). All statistical analyses were performed using the SAS statistical software package (ver. 9.3, SAS Institute, Cary, NC, USA).

## Results

### General characteristics

Table 1 describes the characteristics of study subjects and regions at baseline (*n* = 9,997). A high proportion (54%) of the patients was between 0 and 3 years of age; 57.3% of the patients were males, and 97% were National Health Insurance beneficiaries. In total, 29.4% of patients had a existence comorbidity; 11.1% used an inhaled corticosteroids, and 5.5% exhibited respiratory distress. Of all the children in the sample, 85.7% frequently used the clinic. The mean HHI of the 253 districts including 17,029 hospitals was 1959.5 (standard deviation (SD), 47.7), the mean of number of beds per 1000 population 11.3 (SD, 6.8), the mean of number of doctors per 1000 population 2.3 (SD, 2.0), and the mean of total outpatient health care provider 70.9 (SD, 47.7). Table 2 presents COC distributions and hospital admissions by year. The proportion of hospital admissions ranged between 0.7% and 2.4% and decreased across years; in the group making more than two visits, 55.1% of patients visited a single health care provider or institution in 2009 compared to 14.7% in 2013.

**Table 1:** Baseline characteristics of study subjects.

Characteristics	Study subjects (*N* = 9,997)
**Individual variables**	
**Age (y), *n* (%)**	
0–3	5,458 (54.6)
4–6	3,182 (31.8)
7–9	814 (8.1)
10–12	543 (5.4)
**Sex, *n* (%)**	
Male	5,732 (57.3)
Female	4,265 (42.7)
**Health insurance type, *n* (%)**	
National health insurance	9,701 (97.0)
Medical aid	296 (3.0)
**Residential area**	
Urban	9,462 (94.7)
Rural	535 (5.4)
**The other diseases, *n* (%)**	
No	7,054 (70.6)
Yes	2,943 (29.4)
**Respiratory distress**	
Yes	550 (5.5)
No	9,447 (94.5)
**Use of inhaled corticosteroid**	
Yes	1,112 (11.1)
No	8,885 (88.9)
**Number of total visits**	
1	5,279 (52.8)
2	809 (8.1)
3	649 (6.5)
≥ 4	3,260 (32.6)
**Main type of attending clinic**	
Tertiary general hospital	190 (1.9)
General hospital	591 (5.9)
Hospital	649 (6.5)
Clinic	8,567 (85.7)
Regional variables*	
**HHI**^†^, **mean (SD)**	1959.5(47.7)
**Number of beds per 1,000 population**	11.3(6.8)
**Number of doctors per 1,000 population**	2.3(2.0)
**Total outpatient health care provider**	70.9(47.7)

*, measuring regional variables in 253 administrative districts according to geographical boundaries at the Si, Gun, and Gu (city, county, ward) levels.

^†^, HHI, Herfindahl–Hirschman Index; A low HHI score indicates the presence of less-dominant hospitals, or more competition in that particular market.

**Table 2:** Continuity of care and hospital admissions, by year.

	Study subjects (*N* = 9,997)				
	2009	2010	2011	2012	2013
UPC index* **(mean, SD)**	0.82 (0.14)	0.83 (0.14)	0.85 (0.13)	0.85(0.12)	0.86 (0.11)
**Continuity of care, *n* (%)**					
≥ 4 visits and UPC index (above mean)	4,092 (40.9)	3265 (32.7)	3,369 (33.7)	1,673 (16.7)	927 (9.3)
≥ 4 visits and UPC index (below mean)	655 (6.6)	486 (4.9)	445 (4.5)	213 (2.1)	114 (1.1)
≤ 3 visit	5,293 (52.5)	6,246 (62.5)	6,183 (61.8)	8,111 (81.1)	8,956 (89.6)
**Hospital admission, *n* (%)**					
Yes	243.4)	175 (1.8)	67 (1.7)	69 (0.7)	33 (0.4)
No	9,754 (97.6)	9,797 (98.2)	9,805 (98.3)	9,903 (99.3)	9,939 (99.6)

*, the index ranges between 0–1; a higher value corresponds to improved continuity of care.

### The association between individual and regional variables and COC (UPC index)

Table 3 presents the regression coefficient estimates from the multilevel models. After adjusting for all individual variables which include age, sex, health insurance type, residential area, existence comorbidity, respiratory distress, the use of inhaled corticosteroid, and the main type of attending clinic, and all regional variables which include HHI, the number of beds per 1000 population, the number of doctors per 1000 people, and total outpatient health care provider, when HHI increased by 100,000 points, UPC index scores were also found to increase (*ß* = 0.11, *p* < 0.0001). This indicates that the COC of children living in areas with less market competition was higher. With increasing numbers of doctors per 1,000,000 population, UPC index scores also decreased (*ß* = -0.21, *p* < 0.05). In addition, because of the distribution of UPC and HHI scores, we analyzed using log transformation of UPC and HHI scores, and we presented the results before and after the log transformation.

**Table 3:** Multilevel regression analysis: the associations between HHI and UPC scores (N = 9,997).

	Adjusted^†^ UPC Index			Ln (UPC index)		
	Adjusted *ß*^†^	*(SE*)		Adjusted *ß*^†^,	(*SE*)	
HHI increase by 100,000 points	0.11	0.03	0.22***	0.37^‡^	-0.12	0.45
Total number of hospitals per 100,000 population	0.03	-0.01	0.05	0.06	-0.18	0.06
Number of beds per 1,000,000 population	0.23	-0.06	0.08	0.11	-0.92	1.14
Number of doctors per 1,000,000 population	-0.21	-0.45	-0.04*	-0.29	-0.56	-0.16*

HHI, Herfindahl–Hirschman Index; *SE*, standard error.

*p < 0.05.

***p < 0.0001.

^†^, full model; adjusted for all individual and regional variables.

^‡^, coefficient for Ln (HHI scores).

### Factors associated with hospital admission

Table 4 describes the association between individual characteristics and hospital admissions after controlling for all regional variables which include age, sex, health insurance type, residential area, existence comorbidity, respiratory distress, the use of inhaled corticosteroid, and the main type of attending clinic, and all regional variables which include HHI, the number of beds per 1000 population, the number of doctors per 1000 population and the total outpatient health care provider, evaluated using multilevel logistic regression. The adjusted odds ratio (OR) for children between 0–2 years of age, relative to the reference group (10–12 years of age) was 1.55 (95% CI: 1.15, 2.10). Children who exhibited respiratory distress or were using inhaled corticosteroids were characterized by a higher OR compared to those who did not use corticosteroids (OR, 2.46; 95% CI: 1.77–3.42; and OR, 2.23; 95% CI: 1.84–2.72, respectively). The adjusted OR was 2.72 (95% CI: 2.14–3.46) for those visiting the ambulatory clinic on more than four occasions and with a UPC index with below mean compared to the reference group (≥ 4 ambulatory visits and a UPC index with above mean). The adjusted HHI OR for children residing in quartile 2 was 1.41(95% CI, 1.02–1.94), relative to the HHI values of those in quartile 1. Increases in the number of beds per 1000 population were associated with increased ORs. The adjusted OR for quartile 3 of the number of bed per 1000 population was 1.79 (95% CI, 1.24–2.59), and the adjusted OR for quartile 4 was 1.69 (95% CI, 1.13–2.52), relative to the reference group (quartile 1).

**Table 4:** ORs and 95% CIs for hospital admissions: results of multi-level logistic regression models (N = 9,997).

	Model 1*		Model 2*	
	OR	95% CI	OR	95% CI
***Individual-level variables***				
Age (y; ref. 10–12)				
0–2	1.53	(1.14–2.06)	1.55	(1.15–2.10)
3–5	0.90	(0.67–1.21)	0.91	(0.68–1.22)
6–9	0.86	(0.63–1.16)	0.87	(0.64–1.18)
Sex (ref. Male)				
Female	0.77	(0.65–0.92)	0.77	(0.65–0.91)
Health insurance type (ref. National health insurance)				
Medical aid	1.69	(1.14–2.49)	1.67	(1.13–2.46)
Residential area (ref. Rural)				
Urban	1.06	(0.64–1.78)	1.20	(0.69–2.08)
The other diseases (ref. No)				
Yes	2.42	(1.74–3.36)	2.46	(1.77–3.42)
Respiratory distress (ref. No)				
Yes	3.75	(2.99–4.69)	3.80	(3.03–4.76)
Use of inhaled corticosteroid (ref. No)				
Yes	2.24	(1.84–2.72)	2.23	(1.84–2.72)
Total number of visits (ref. 1)				
2	0.81	(0.58–1.13)	0.81	(0.58–1.13)
3	0.84	(0.58–1.20)	0.83	(0.58–1.20)
≥ 4	1.15	(0.93–1.43)	1.15	(0.92–1.43)
Main clinic type attended (ref. Clinic)				
Tertiary general hospital	6.50	(4.57–9.23)	6.57	(4.60–9.38)
General hospital	7.22	(5.73–9.09)	7.20	(5.72–9.07)
Hospital	4.00	(3.06–5.24)	3.96	(3.03–5.19)
Continuity of care (ref. ≥ 4 visits and UPC index with above mean)				
≥ 4 visits and UPC index with below mean	2.66	(2.09–3.39)	2.72	(2.14–3.46)
≤ 3 visit	0.69	(0.59–0.99)	0.69	(0.57–0.84)
***Region-level variables***				
HHI (ref. Quartile 1)				
Quartile 2			1.41	(1.02–1.94)
Quartile 3			1.08	(0.74–1.57)
Quartile 4			0.97	(0.63–1.49)
Number of hospitals per 1,000 population (ref. Quartile 1)				
Quartile 2			0.90	(0.65–1.25)
Quartile 3			0.84	(0.58–1.23)
Quartile 4			0.70	(0.45–1.07)
Number of bed per 1,000 population (ref. Quartile 1)				
Quartile 2			1.14	(0.81–1.60)
Quartile 3			1.79	(1.24–2.59)
Quartile 4			1.69	(1.13–2.52)
Number of doctors per 1,000 population (ref. 1)				
2			0.90	(0.60–1.34)
3			0.95	(0.57–1.56)
≥4			0.94	(0.51–1.71)

*, In Model 1, results were adjusted for all individual variables; in Model 2, results were adjusted for all individual and regional variables.

## Discussion

We examined the relationship between market competition and COC, and assessed the effect of COC on hospital admissions in asthmatic children. Children living in less competitive areas were characterized by a higher UPC index; those with a UPC index score of 1 exhibited a lower OR for hospital admission. The ORs of children visiting more than one health care provider or institution was 3.61-fold higher compared to those who visited only one health care provider or institution.

The COC of children living in more competitive areas was lower; a low COC was associated with a higher OR for hospital admission. At the same time, the adjusted odds ratio of hospital admission for groups in quartile 1 regarding HHI was greater relative to the group in quartile 2 regarding HHI. Namely, the odds ratio for hospital admission may be the higher when the level of competition is lower. Korea’s health care market lies on competition among health care providers. This relates to health care delivery system, which is organized as follows: clinics function as primary care institutions, hospitals functions as secondary care institutions, and general hospitals and tertiary general hospitals function as tertiary care institutions [27]. As mentioned above, all of these institutions can provide outpatient services with no barriers to the use of superior health services. Furthermore, in Korea, 91% of clinical physicians are specialists, and 98% of clinics were the sole practice used during 2006 [28]. Many previous studies have shown that higher market competition may improve quality of care as a whole [20]. However, when concerning individual aspects, patients had more opportunity to select healthcare providers and was able to more easily access suitably qualified physicians when higher levels of competition was present among health care providers and patients had more freedom in selecting health care providers. Moreover, unalike adult patients, if the symptoms of asthmatic children did not improve speedily, parents were willing to seek a different health care provider. These circumstances including high market competition may lead to a reduction in the continuity of care and there may be disadvantages in the management of patient information. As a result, children with low COC may be likely to admitted.

Our data indicate that superior COC is associated with improved health outcomes, which is consistent with previous studies. However, we could not identify the mechanism underlying this association. Gray et al. (2003) suggested that COC could benefit patients by reinforcing the accumulation of patient knowledge, improving interpersonal communication, and increasing the likelihood that patients adhere to their doctor’s advice. In addition, our findings reveal that higher number of hospitals per 1000 population is also associated with higher UPC but that higher numbers of physician per 1000 population is associated with lower UPC scores. The feature shared by most small areas is that there is a lack of general hospitals and tertiary hospitals with a sufficient number of physicians. In fact, even if a certain district has many hospitals, if all of these hospitals are institutions that provide medical services through one physician, the district will be score high in the total number of hospitals per 1,000 population but will score low in the number of doctors per 1,000 population. In contrast, if a certain district has few hospitals but several of these hospitals are general hospitals or tertiary hospitals, the district will score low in the total number of hospitals per 1000 population and will score high in the number of doctors per 1000 population. Therefore, the total number of hospitals and number of doctors per 1000 population depends on the composition of general hospitals, which include many physicians and clinics operated solely by one physician. Differences in this composition in each district may lead to the shown results.

Patients receiving care at general/tertiary hospitals were more likely to be admitted than those treated at clinics. Although we attempted to adjust case complexity and disease severity according to respiratory distress status and CCI scores, our data appear to reflect the fact that disease severity is greater in patients using higher-level hospitals. Although several previous studies reported that the use of inhaled corticosteroid is associated with a reduced likelihood of hospital admission and use of emergency medical services [29–31], our data indicated the opposite. The majority of our sample principally used clinics for outpatient care. In October 2010, the Korean incentive system for outpatient, drug-focused clinics was introduced to reduce drug and prescription costs [32]. Inhaled corticosteroids are more expensive compared to oral corticosteroids; physicians are therefore more likely to prescribe oral corticosteroids, and only to children with severe asthma. This situation may have influenced our equivocal result.

This study had several limitations. First, with regard to the accuracy of the asthma diagnosis, we used the National Health Insurance claims database from HIRA. The claimed data are sent to HIRA, which reviews the claims for reimbursement. The compulsory nature of the Korean NHI system (established in 1989) means that the system provides universal coverage. It was one of the earliest adaptive organizations to exercise leadership in information technology (IT) among public institutions by strengthening and providing coverage using the latest IT[33]. However, this can serve as limitations in the use of these databases. These data do not include uninsured events and only represent services covered by the NHI. The data may not be consistent with actual diagnosis because it is ordinarily made for claiming purposes. Diagnostic accuracy in KNHI claims data are approximately 70%[34]; to increase accuracy, we reviewed all prescriptions and diagnostic tests, but 100% accuracy could nonetheless not be guaranteed. In addition, 94.5% of the subjects had no respiratory distress. We defined respiratory distress as having an ICD-10 code of R06. R06.0 is dyspnea, R06.1 is stridor, and R06.2 is wheezing. When physicians conducted physical examinations, respiratory distress might not have been present or physicians may not have conducted coding. This phenomenon may lead act as a limitation of claims data. Second, we could not consider all factors that might affect the association between UPC index scores and hospital admissions, such as income level, education, physician characteristics, and general health behaviors, because this information was not present in the claims database. Third, we could not assess the quality of outpatient clinics according to the degree of market competition. Finally, we could not identify individual service providers on the basis of information extracted from the claims database. Therefore, outpatient healthcare providers were medical institutions rather than physicians.

Despite these limitations, our study also had several strengths. First, we analyzed a representative sample of asthma patients using nationwide claims data and a longitudinal design. In addition, we adjusted for disease severity by recording respiratory distress status and including prescription of asthma medication as a criterion of ambulatory care. Finally, we considered both individual and regional variables that may be associated with COC and hospital admission rates.

While market competition may help improve quality of care, it may also induce patients’ discontinuity of care. Especially, these phenomena get worse in countries like Korea, which provide universal health insurance coverage, and where all health care providers compete with one another and patients can select any health care institution without certain regulation such as gatekeeper. Therefore, we need to reform health care delivery system to prevent unnecessary hospitalization and reduce costs due to discontinuity. Also, market competition should be managed while improving quality of care through an accountable care organization (ACO), which accepts financial responsibility, care accountability and at the same time increases value in the health care system by reducing cost without worsening quality [35].

In conclusion, our results support the hypothesis that improving COC can reduce hospital admission risk in asthmatic patients. We encourage policymakers to recognize the need for an effective healthcare delivery system promoting COC, by encouraging a collaborative approach, enhancing accessibility, and considering the impact of market competition.

## Supporting Information

S1 Text About the all the relevant data of our study data. (DOCX)
