# Supplementary material for: Impact of Market Competition on Continuity of Care and Hospital Admissions for Asthmatic Children: A Longitudinal Analysis of Nationwide Health Insurance Data 2009-2013
Source: PLoS One. 2016 Mar 9;11(3):e0150926. doi: 10.1371/journal.pone.0150926 (PMC4795663; doi:10.1371/journal.pone.0150926)
Supplement: S1 Text — (DOCX) [file pone.0150926.s001.docx]

**About the all the relevant data of our study data**

Our data was not available. We obtained from YS, Nam. YS, Nam is working at HIRA as a researcher. And because he is an internal researcher at HIRA, HIRA only provided him with the necessary data. Hence our data was not secondary data released to general researchers and is not available to all general researchers.

Our data is available from Health Insurance Review & Assessment, researchers can obtain through administration process after filling out documents. Our data is not ethically restricted. Because our data is not including personal information, and our data is released for study.
